# Tip‐to‐base conduit widening maintains hydraulic efficiency in aerial fern organs

**DOI:** 10.1111/nph.70467

**Published:** 2025-08-12

**Authors:** Yuliia Khoma, Scott A. M. McAdam

**Affiliations:** ^1^ Department of Botany and Plant Pathology Purdue University West Lafayette IN 47907 USA; ^2^ Institute of Cell Biology and Genetic Engineering National Academy of Sciences of Ukraine Kyiv 03143 Ukraine

**Keywords:** fern, hydraulic diameter (*Dh*), tapering, turgor, water potential, xylem

## Abstract

- Tip‐to‐base conduit widening is critical for hydraulic efficiency; yet few studies have investigated the full developmental potential of xylem.
- We utilized the diverse growth habits of ferns to test whether a wide developmental potential of xylem is constrained by growth habit. We documented xylem hydraulically weighted conduit diameter (*Dh*) across the vertical stem of the tall *Equisetum giganteum* L.; in the soil surface rhizome that roots at each node of *Phlebodium pseudoaureum* (Cav.) Lellinger and its vertical leaf. In addition, we determined *Dh* across aquatic and aerially suspended rhizomes of *Marsilea hirsuta* L.
- Considerable tip‐to‐base conduit widening was observed in the stems of *E. giganteum*, aerially‐suspended rhizomes of *M. hirsuta*, and the vertical leaves of *P. pseudoaureum*, organs in which selection would favor the maintenance of hydraulic efficiency toward the apex. No change in *Dh* was observed along the soil‐surface rhizomes of *P. pseudoaureum* or the aquatic rhizomes of *M. hirsuta*.
- Our results indicate that there is considerable developmental potential for xylem conduit diameter in ferns apparent under conditions in which hydraulic efficiency is not required for survival; but that tip‐to‐base conduit widening is consistently observed in fern organs that require an efficient long‐distance transport of water to the apex.

## Анотація

- Розширення провідних елементів ксилеми від верхівки до основи є критично важливим для гідравлічної ефективності, однак незначна кількість досліджень присвячено вивченню повного потенціалу розвитку ксилеми.
- Ми використовували різноманітні типи росту папоротей, щоб перевірити, чи обмежується широкий потенціал розвитку ксилеми способом росту. Для цього ми визначали гідравлічно зважений діаметр провідних елементів (*Dh*) уздовж: вертикального стебла *Equisetum giganteum* L.; вертикального листка та наземного кореневища *Phlebodium pseudoaureum* (Cav.) Lellinger, що стелиться по поверхні ґрунту й укорінюється у вузлах, а також уздовж водних і підвішених у повітрі кореневищ *Marsilea hirsuta* L.
- Значне розширення провідних елементів ксилеми від верхівки до основи було зафіксоване у стеблах *E. giganteum*, повітряних кореневищах *M. hirsuta* та вертикальних листках *P. pseudoaureum* — органах, в яких природний добір міг би сприяти підтриманню гідравлічної ефективності у напрямку до апексу. Натомість, жодних змін у *Dh* не спостерігалось уздовж наземних кореневищ *P. pseudoaureum* чи водних кореневищ *M. hirsuta*.
- Наші результати свідчать про те, що папороті мають значний потенціал розвитку діаметра провідних елементів ксилеми, який реалізується за умов, коли гідравлічна ефективність не є критичною для виживання. Проте, стабільне розширення провідних елементів від верхівки до основи спостерігається в тих органах папоротей, які потребують ефективного довготривалого транспорту води до апексу.

## Introduction

Vascular plants efficiently transport water and dissolved nutrients from the roots to other organs using a specialized tissue called xylem. Being largely comprised of dead sclerenchyma cells, xylem provides a low resistance transport path for water, which is relatively resistant to embolism formation and provides mechanical strength (Baas *et al*., 2004; Chave *et al*., 2009; Brodribb & Mencuccini, 2017). The morphological characteristics of xylem elements have considerable influence over water transport efficiency and safety. Of particularly note, across the stems of terrestrial plants, xylem anatomy varies in a highly consistent way, with conduits widening from tip‐to‐base, sometimes this phenomenon is also referred to as conduit tapering (Sanio, 1872; Rosell *et al*., 2017; Lechthaler *et al*., 2020; Olson *et al*., 2021; Chambers‐Ostler *et al*., 2023). This near‐universal observation has important adaptive explanations (Petit, 2024). It has been proposed that as plants grow taller, the diameter of the distal xylem vessels must increase to reduce the resistance to water flow and improve the efficiency of water transport throughout the plant (West *et al*., 1997; Olson & Arroyo‐Santos, 2015; Koçillari *et al*., 2021).

The observation of tip‐to‐base conduit widening across a wide diversity of terrestrial plant species, following a power‐function with an exponent of *c*. 0.2, suggests this trait is under strong selection (West *et al*., 1999; Becker *et al*., 2000; Anfodillo *et al*., 2006; Koçillari *et al*., 2021; Bok *et al*., 2022). Tip‐to‐base conduit widening as an adaptation, favored by selection, is currently the best‐supported model explaining this trait because of the need to maintain conductance as the conductive pathlength and hydraulic resistance increases (Olson *et al*., 2021; Rodriguez‐Zaccaro *et al*., 2021). Evidence supporting a strong selective pressure driving a consistent developmental tip‐to‐base widening of conduits arises from studies at the population level (Rodriguez‐Zaccaro *et al*., 2021), interspecific comparisons (Olson *et al*., 2018, 2021), and optimality modeling (West *et al*., 1999; Petit & Anfodillo, 2009; Savage *et al*., 2010; Couvreur *et al*., 2018; Koçillari *et al*., 2021).

A less‐likely alternative, given how common optimality models predict tip‐to‐base conduit widening across the diversity of land plants, is the hypothesis that the widening of xylem conduit diameter away from the apex is really a tapering towards the apex that is driven by hydraulic constraints on plant development (Cabon *et al*., 2020). High turgor pressure is required for cell expansion, which may affect the diameter of xylem cells. Given the gradient in water potential across a stem from the soil to the sites of evaporation in the leaf, there is a natural gradient in water status and turgor pressure along a stem, which might result in reduced developmental expansion of vessels (Ray & Green, 1972; Hacke *et al*., 2006; Cabon *et al*., 2020). If passive cell expansion, driven by turgor, is the primary mechanism for conduit tapering, then it is less likely that this phenomenon is under active selection for increased efficiency in water transport. Instead, it could simply be a function of plant water status during cell expansion. Consequently, factors such as drought, high temperature, or increased evaporative demand might influence the extent of conduit tapering (Sperry *et al*., 2002; Tyree & Zimmermann, 2002; Sack & Holbrook, 2006; Meinzer *et al*., 2010; Anfodillo *et al*., 2012; Lens *et al*., 2013; Rosell & Olson, 2014).

Most data to date documenting xylem tip‐to‐base conduit widening derives from terrestrial plants in which strong selection should be driving a consistent exponential relationship between conduit diameter and proximity to stem apex (Koçillari *et al*., 2021). We do not know if selection for tip‐to‐base conduit widening is relaxed under aquatic conditions, or conditions in which there is no gradient of water status along the stem. Drobnitch *et al*. (2015) have shown that there is phloem conduit tapering in kelp, suggesting that similar tip‐to‐base conduit scaling might occur in all plants, not just terrestrial species.

We hypothesized that given the observations of tip‐to‐base conduit widening across terrestrial plants (Anfodillo *et al*., 2006; Olson *et al*., 2014; Koçillari *et al*., 2021; Bok *et al*., 2022), we would observe this phenomenon broadly across ferns. The structure of xylem in ferns differs significantly from most seed plants, as it is comprised of primary xylem, which does not undergo secondary growth (Suissa & Friedman, 2021). The primary elements of the xylem in most species of ferns are tracheids (Mahley *et al*., 2018), which are elongated cells with thickened lignified walls, but vessels have been reported in a number of groups, including the genus *Marsilea* (Schneider & Carlquist, 2000b). Given the primary nature of fern xylem, it is consequently established early in stem development (Bierhorst, 1960; White, 1963; Carlquist & Schneider, 2001). Another feature of ferns as a group is that there is considerable diversity in stem morphology and growth habit, from near‐arborescent species with erect stems (Smith *et al*., 2008) to terrestrial and epiphytic species that have rhizomes in contact with the substrate, to aquatic and semi‐aquatic species that can have rhizomes that grow in and out of water (Smith *et al*., 2008; Westbrook & McAdam, 2021). Despite this diversity and the long evolutionary history and ecological breadth of this group of land plants, little is known about the vascular properties of xylem of ferns (Pittermann *et al*., 2013; Suissa & Friedman, 2021).

Here we use the diversity of fern stem morphologies to specifically investigate whether tip‐to‐base conduit widening is under selection in this group. Koçillari *et al*. (2021) have previously observed tip‐to‐base conduit widening in the vertical stems of 5.3 m tall *Equisetum myriochaetum* Schltdl. & Cham. and *Selaginella* and *Lycopodium*, as well as in the leaves of *Lophosoria quadripinnata* (J.F.Gmel.) C.Chr. We hypothesize that when stems or leaves of ferns are grown under conditions that require the development of an efficient water transport system, such as in erect aerial stems, rhizomes growing suspended in air out of water or across large leaves, then tip‐to‐base conduit widening will be observed (Koçillari *et al*., 2021; Olson *et al*., 2021). By contrast, when stems are grown under conditions in which there is no environmental pressure to maintain tip‐to‐base conduit widening for hydraulic efficiency, like in prostrate rhizomes that root at each node or in submerged aquatic rhizomes, then alternative conduit scaling functions from tip‐to‐base may manifest.

## Materials and Methods

### Plant material and growth conditions

We chose three species of fern (*Marsilea hirsuta* L., *Equisetum giganteum* L. and *Phlebodium pseudoaureum* (Cav.) Lellinger) that have highly divergent stem or rhizome morphology and habit. Plants were grown in the Lilly glasshouses at Purdue University (West Lafayette, IN, USA).

*Marsilea hirsuta* belongs to a small family of semi‐aquatic ferns, Marsileaceae. *Marsilea* species have a number of highly divergent reproductive, physiological, and ecological traits compared to other fern groups (Schneider & Pryer, 2002; Westbrook & McAdam, 2021). *Marsilea hirsuta* has a rhizome capable of transitioning between aquatic and terrestrial environments (Westbrook & McAdam, 2021). The rhizomes are typically slender and creeping, allowing the plant to root both underwater and in moist ground. They have compound leaves with four leaflets that are highly responsive to environmental signals, possessing nyctinasty (daily movement of leaflet orientation), a trait not found in other fern groups (Vasco *et al*., 2013). Plants of *M. hirsuta* were grown in flooded tubs on a bench. The tubs were half‐filled with topsoil; plants were allowed to grow to fill the tub, and rhizomes were allowed to extend and grow over the edge of the tub so that they became suspended in air.

*Equisetum giganteum* is a giant horsetail, one of the largest living members of this group of ferns (Husby, 2009). *Equisetum giganteum* has a hollow upright stem, reinforced by nodal thickenings and septa, and can grow to over 5 m in height. The relatively small diameters of *E. giganteum* stems (< 4 cm) pose a mechanical stability challenge against buckling (Husby, 2009).

*Phlebodium pseudoaureum* is a terrestrial or epiphytic fern that has large glaucous fronds (up to 1 m in length) originating from a broad, trichome‐covered, prostrate rhizome that clings to the soil surface and roots at each node (Tejero‐Díez *et al*., 2009). Mature individuals of *E. giganteum* and *P. pseudoaureum* were grown in in‐ground beds in the glasshouse with no restriction on growth space or light.

### Sample preparation and anatomical measurements

Three rhizomes, stems, or leaves were selected for each fern species or growth type in *M. hirsuta*. Distance from the apex was measured in all. Transverse sections along the rhizomes of *M. hirsut*a were made every 1 cm from the apex for 35–40 cm. In *P. pseudoaureum*, transverse sections of the rhizome from the apex were made every 10 cm for 40 cm, as well as across a frond from leaf tip to base at the node. Since the length of the stem in *E. giganteum* exceeded 2 m, we made transverse sections along the length of the stem every 40 cm; we found that xylem had not fully developed and matured in the upper 40 cm of the tall stems. Conduit data collected in this cell differentiation and elongation region was not used to determine conduit scaling relationships. Cross‐sections (20–30 μm thick) were prepared using a freezing microtome (Microm HM 430; Thermo Scientific, Waltham, MA, USA). Sections were stained with aqueous toluidine blue (0.1% in H_2_O) before being mounted on a glass slide using glycerine jelly. Stained sections were examined using a fluorescence microscope (Zeiss Axio Scope) with an appropriate long pass filter for autofluorescence, and digital images of the xylem were taken under fluorescence at ×10 magnification for *M. hirsuta* and under visible light at ×10 magnification for rhizomes of *P. pseudoaureum* and at ×20 magnification for *E. giganteum* samples. Measurements of xylem conduit diameter were conducted using ImageJ (Schindelin *et al*., 2012).

Conduit diameter was calculated assuming the lumen to be circular. We chose conduits with a diameter of more than half the diameter of the largest conduit to eliminate those that may have been tapered ends, as suggested by James *et al*. (2003). Selected cells were averaged as the hydraulic diameter (*Dh*), so that cell diameters were weighted according to hydraulic conductance (Kolb & Sperry, 1999; Anfodillo *et al*., 2006):

$$\mathrm{Dh}=\frac{\sum d_{n}^{5}}{\sum d_{n}^{4}}$$

where *d*_*n*_ is the diameter of the *n* cell (Sperry *et al*., 1994).

### Water potential

Three leaves, or stem apices, from each species were collected from across the length of the rhizome (apex, middle and base‐ which we considered to be the end of the length along which conduit diameter was measured) along the rhizome of each fern sample for determination of mean midday leaf water potential (MPa) on a sunny day. Leaves were excised and wrapped in damp paper towel with no surface water and immediately placed into a humid plastic bag as per Rodriguez‐Dominguez *et al*. (2022). Midday leaf water potential was measured using a pressure chamber (PMS Instrument Co., Corvallis, OR, USA). Stem water potential was measured in *E. giganteum* by sampling shoots pre‐dawn from across the canopy.

### Statistical analyses

Statistical analysis was performed according to common methods. Data are presented as the mean value ± SE. Differences between xylem hydraulic diameter measurements along the rhizome from tip to base and water potential were analyzed using a one‐way analysis of variance (ANOVA). Mean comparisons were performed using a *post‐hoc* pairwise Tukey's test. All tests were declared significant at *P* < 0.05. Statistical analyses were performed in the SPSS 29.0 Software (IBM, Inc., NY, USA).

## Results

### Anatomical measurements

Mean hydraulic diameter of xylem conduits (*Dh*) along the leaf, rhizome, or stem varied across the three species of ferns examined (Table 1). *M. hirsuta* had the narrowest conduit diameter of all species measured, with stems growing in water having a mean *Dh* of *c*. 54.03 ± 2.27 μm (Table 1). In *E. giganteum*, the largest mean *Dh* was 62.77 ± 0.43 μm at the base of the stem (Table 1). In *P. pseudoaureum*, the largest mean *Dh* was 85.34 ± 1.79 μm measured in the rhizome, with the mean *Dh* at the base of the stipe being 76.13 ± 9.48 μm narrowing to 25.07 ± 4.51 μm in the midrib at the leaf apex (Table 1).

**Table 1:** Average hydraulic diameter (*Dh*) of xylem from top to base in ferns rhizomes, stems or leaves.

Species	Organ	Distance from apex (cm)	Dh (μm) ± SE	Exponent
*Marsilea hirsuta*	Aquatic rhizome	1	53.00 ± 1.73^a^	0
		15	54.03 ± 2.27^a^	
		30	54.78 ± 2.03^a^	
*Marsilea hirsuta*	Aerially suspended rhizome	1	41.99 ± 1.27^a^	0.27
		20	49.53 ± 1.83^b^	
		45	59.60 ± 2.46^c^	
*Equisetum giganteum*	Stem	1	35.22 ± 2.27^a^	0.34
		75	46.82 ± 3.044^b^	
		200	62.77 ± 0.43^c^	
*Phlebodium pseudoaureum*	Rhizome	0	83.07 ± 3.33^a^	0
		20	85.34 ± 1.79^a^	
		40	82.28 ± 2.18^a^	
*Phlebodium pseudoaureum*	Leaf	5	25.07 ± 4.51^a^	0.46
		30	54.43 ± 12.66^b^	
		60	76.13 ± 9.48^c^	

Different letters indicate significant differences in *Dh* across a rhizome or stem using a *post‐hoc* pairwise Tukey's test at *P* < 0.05. The exponents for two‐factor power functions fitted to *Dh* and distance from apex data are shown.

**Fig. 1:**
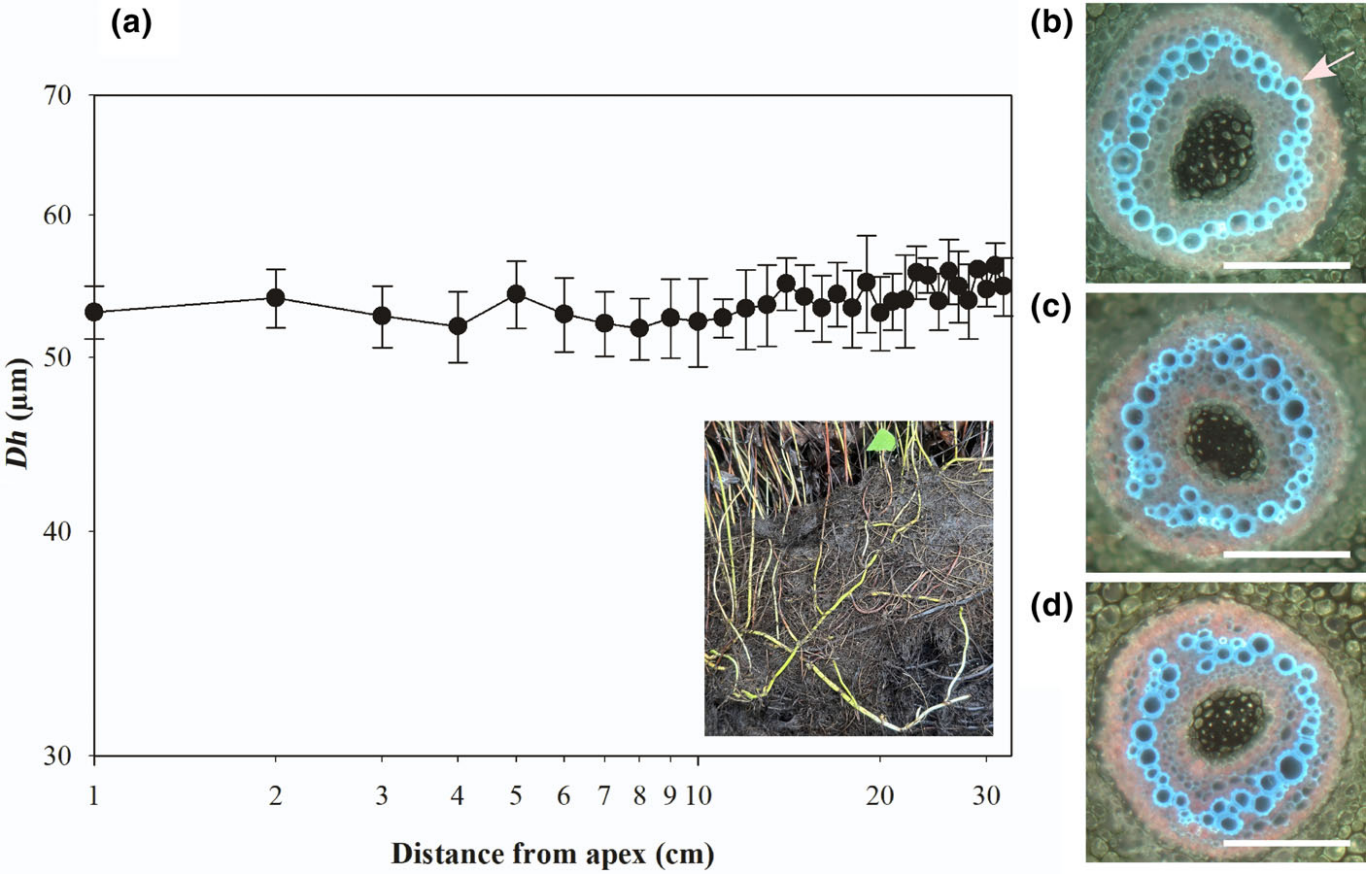
No tip‐to‐base conduit widening in aquatic rhizomes of *Marsilea hirsuta*. (a) Mean hydraulically weighted xylem conduit diameter (*Dh*) along the rhizome of aquatic rhizomes of *M. hirsuta* (insert depicts an aquatic rhizome once water is drained away growing on substrate). Representative images of transverse sections of an aquatic rhizome of *M. hirsuta* (b) at the apex, (c) 15 cm from the apex, and (d) 30 cm from the apex. Sections were stained with aqueous toluidine blue, and images were taken at ×10 magnification under ultraviolet light with a long pass filter. The arrow in b shows the location of the xylem. Bars, 500 μm. Error bars show ±SE (*n* = 3).

**Fig. 2:**
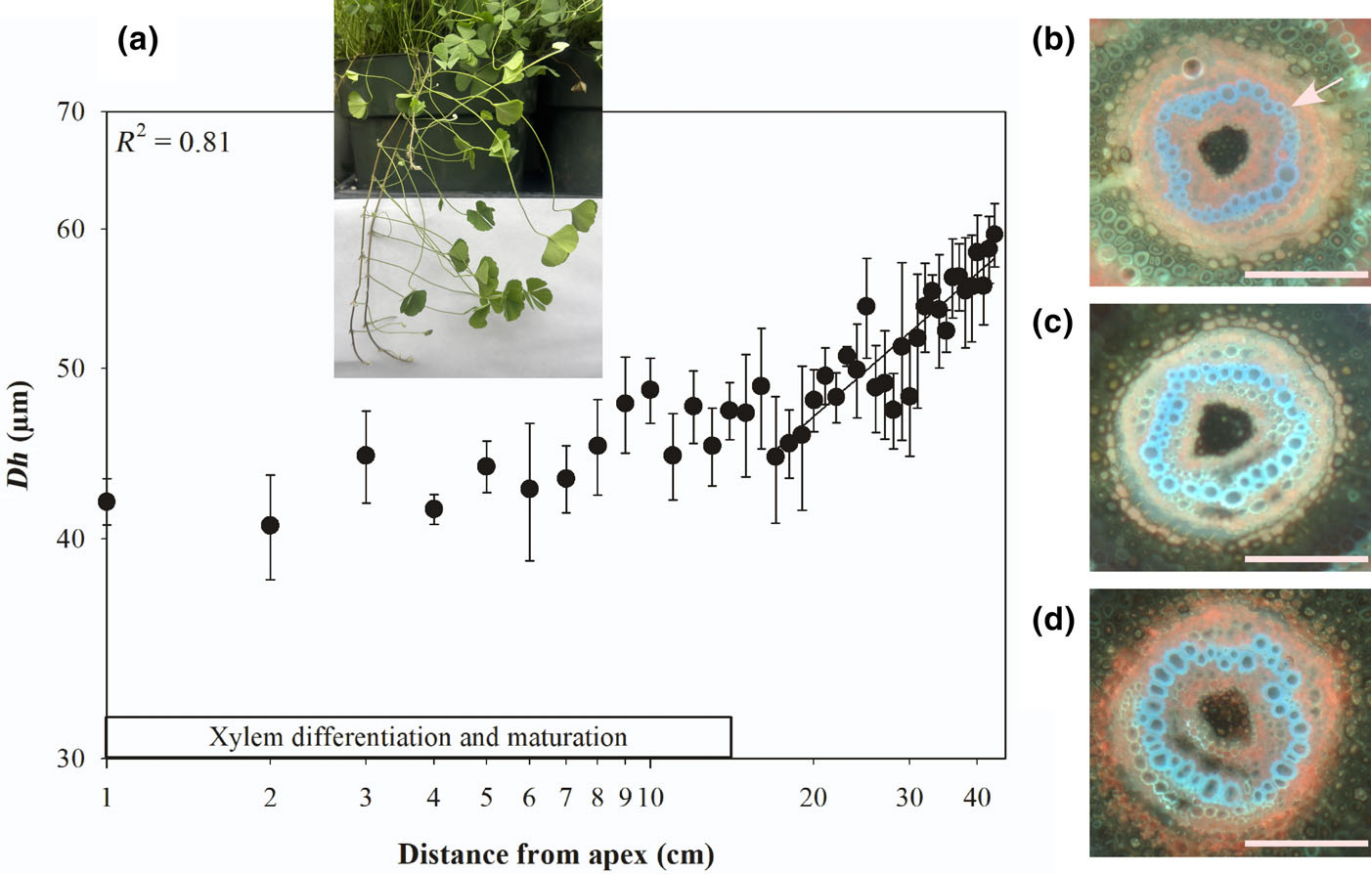
Tip‐to‐base conduit widening in aerially suspended rhizomes of *Marsilea hirsuta*. (a) Mean hydraulically weighted xylem conduit diameter (*Dh*) along aerially suspended rhizomes of *M. hirsuta* that grew away from standing water into air (insert depicts an aerially growing rhizome; bar denotes the region of the rhizome in which active xylem differentiation and maturation is occurring). A two‐parameter power function is fitted to data along the rhizome from when xylem maturation is complete; an *R* ^2^ for this function is shown. Representative images of transverse sections of an aerially suspended rhizome of *M. hirsuta* that grew away from standing water (b) at the apex, (c) 20 cm from the apex, and (d) 42 cm from the apex in close proximity to water. Sections were stained with aqueous toluidine blue, and images were taken at ×10 magnification under ultraviolet light with a long pass filter. The arrow in b shows the location of the xylem. Bars, 500 μm. Error bars show ±SE (*n* = 3). In aerially suspended rhizomes of *M. hirsuta*, the apex was the most distal tissue from the water, more distal than any leaf along the rhizome.

In *M. hirsuta*, *Dh* along aquatic, submerged rhizomes did not change from tip‐to‐base, with mean *Dh* varying between 53 and 55 μm along 30 cm of rhizome (Fig. 1a). By contrast, in rhizomes of this species that grew out of standing water and into air, we observed a considerable and significant reduction in *Dh* towards the apex in regions of the rhizome with mature and no longer differentiating xylem, with xylem maturation and full leaf expansion occurring at *c*. 17 cm from the apex (Fig. 2a). In the aerially suspended rhizomes of *M. hirsuta* growing out of water, *Dh* increased away from the tip (41.99 μm) to base (59.60 μm) (Table 1) 40 cm away from the apex. The rhizome bases were submerged in these aerially suspended stems. This tip‐to‐base widening followed a significant two‐parameter power function (*R* ^2^ = 0.81, *P* < 0.01) with an exponent of 0.27 (Fig. 2a; Table 1).

**Fig. 3:**
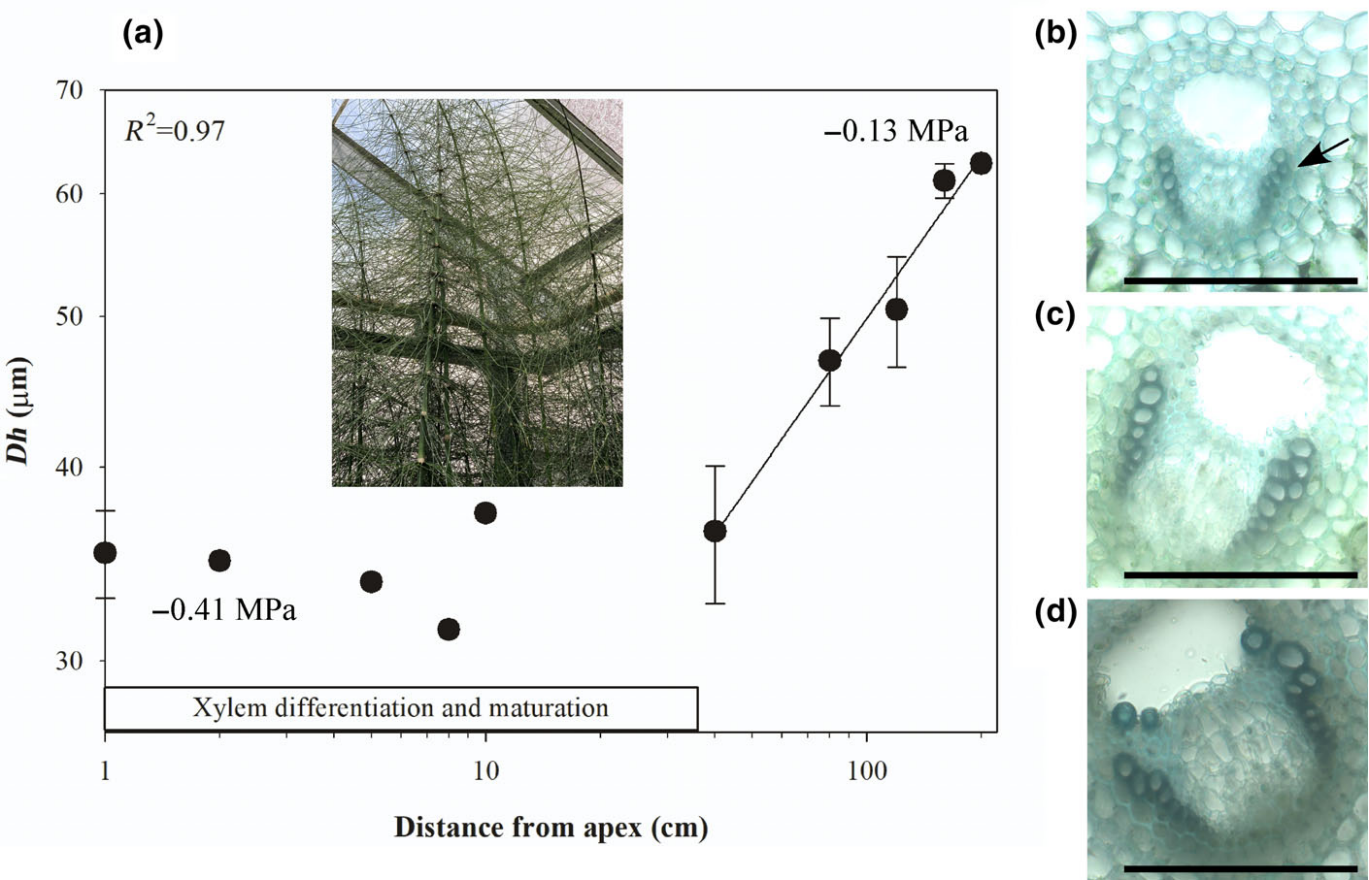
Tip‐to‐base conduit widening in vertical stems of *Equisetum giganteum*. (a) Mean hydraulically weighted xylem conduit diameter (*Dh*) along the vertical stems of *E. giganteum* (the insert depicts the stems of *E. giganteum*; bar denotes the region of the stem in which active xylem differentiation and maturation is occurring). A two‐parameter power function is fitted to data along the rhizome from when xylem maturation is complete; an *R* ^2^ for this function is shown. Mean pre‐dawn stem water potentials at the apex and 150 cm from the apex are given. Representative images of transverse sections of a vascular bundle in the stem of *E. giganteum* at (b) the apex, (c) 120 cm from the apex, and (d) 200 cm from the apex. Sections were stained with aqueous toluidine blue, and images were taken under visible light at ×20 magnification. The arrow in b shows the location of the xylem. Bars, 250 μm. Error bars show ±SE (*n* = 3).

Anatomical measurements of the stem of *E. giganteum* revealed a significant difference in *Dh* relative to the distance from the stem apex (Fig. 3a). *Dh* almost doubled along the stem away from the apex, with *Dh* increasing from a mean of 35.22 ± 2.27 μm once xylem had finished differentiating at *c*. 40 cm from the apex to a mean of 62.77 ± 0.43 μm 200 cm along the stem (Table 1; Fig. 3a). The increase in *Dh* from the tip followed a significant two‐parameter power function (*R* ^2^ = 0.97, *P* < 0.001) with an exponent of 0.34 (Fig. 3a; Table 1).

**Fig. 4:**
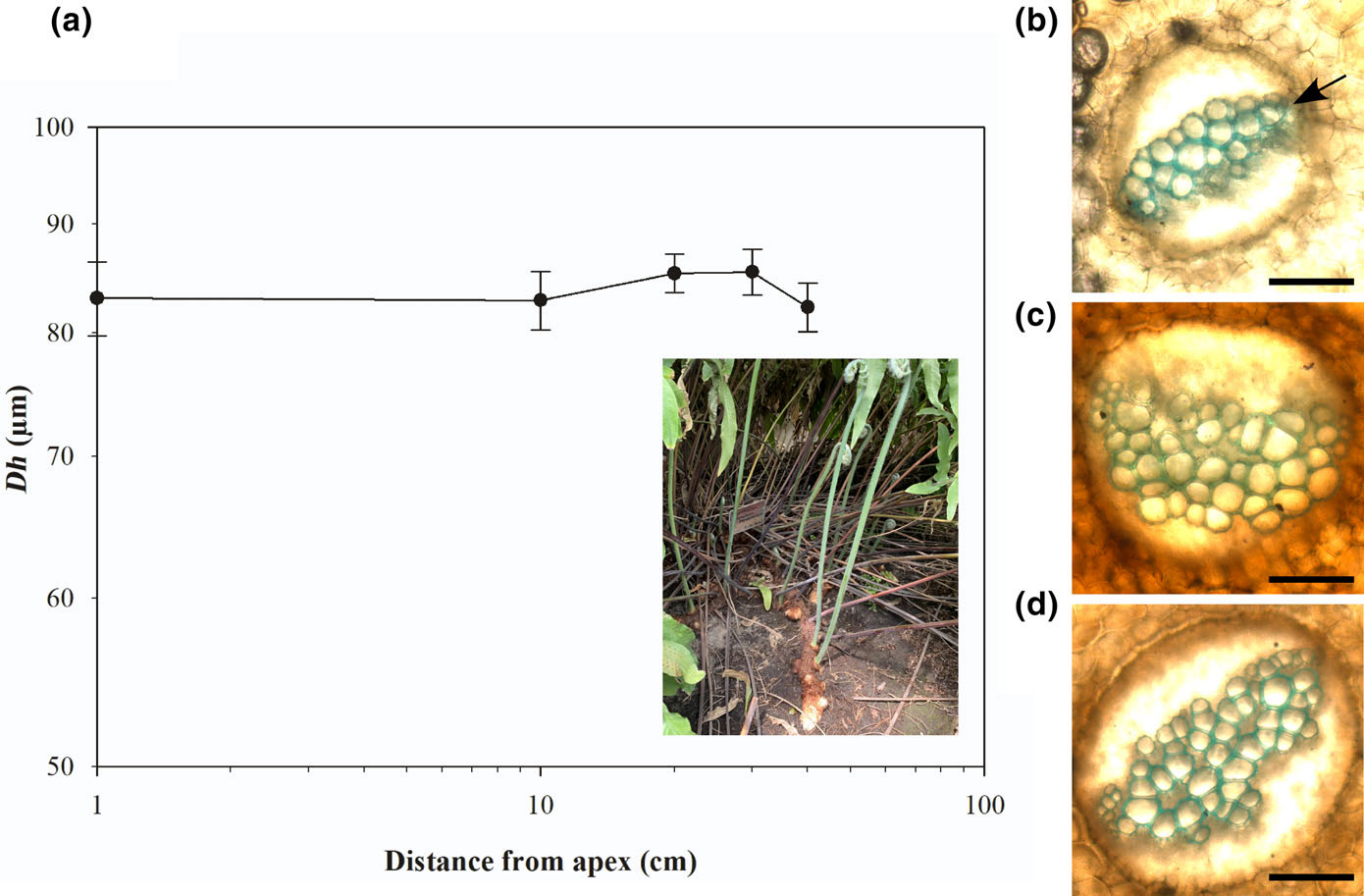
No tip‐to‐base conduit widening in the rhizomes of *Phlebodium pseudoaureum*. (a) Mean hydraulically weighted xylem conduit diameter (*Dh*) along the terrestrially growing rhizome of *P. pseudoaureum*; the insert depicts the rhizome of *P. pseudoaureum*. Representative images of transverse sections of the vascular bundle in the rhizome of *P. pseudoaureum* at (b) the apex, (c) 20 cm from the apex, and (d) 40 cm from the apex. Sections were stained with aqueous toluidine blue, and images were taken under visible light at ×20 magnification. The arrow in b shows the location of the xylem. Bars, 500 μm. Error bars show ±SE (*n* = 3).

**Fig. 5:**
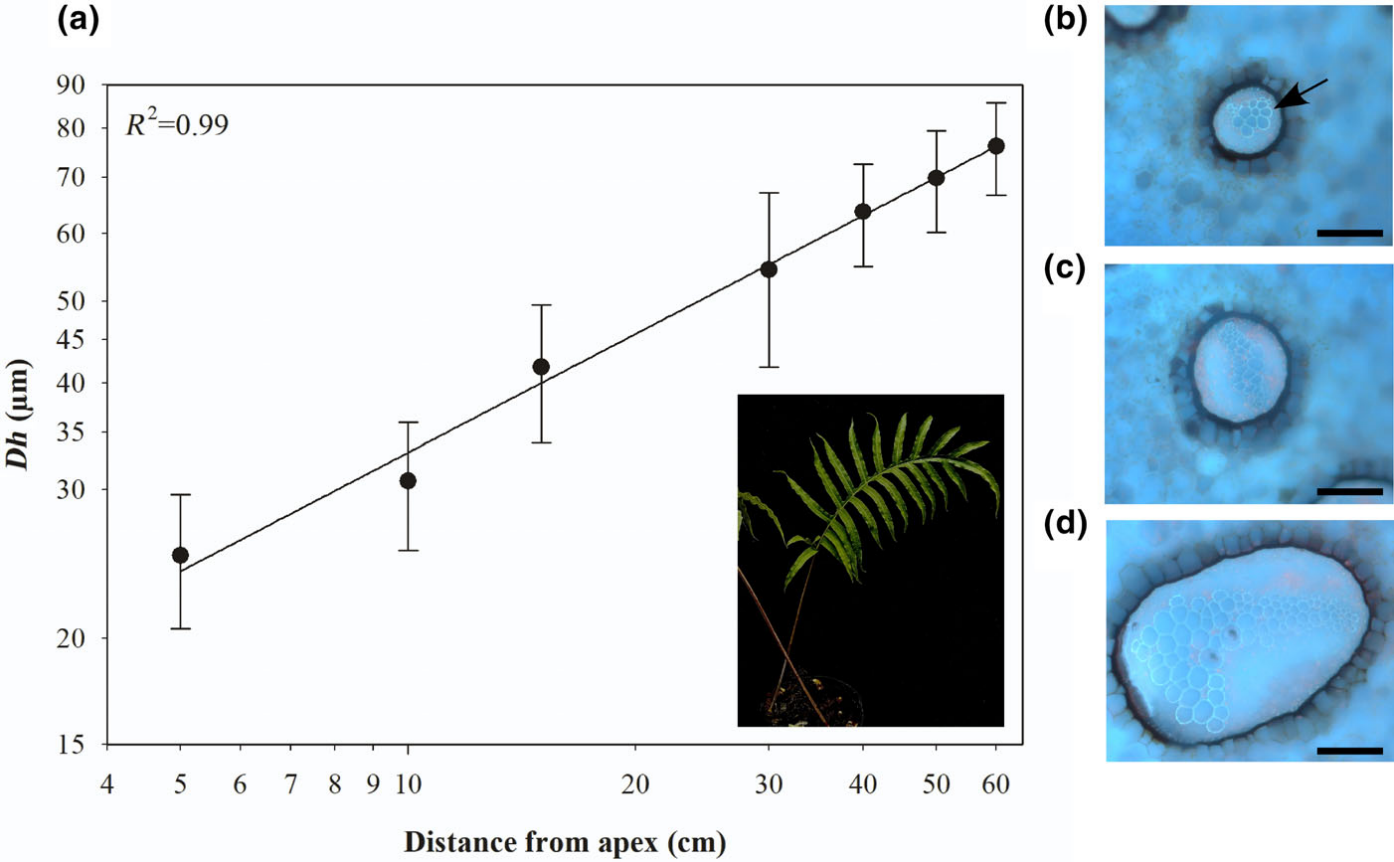
Tip‐to‐base conduit widening across the frond of *Phlebodium pseudoaureum*. (a) Mean hydraulically weighted xylem conduit diameter (*Dh*) along the frond of *P. pseudoaureum*; the insert depicts the frond of *P. pseudoaureum*. A two‐parameter power function is fitted to the data, and an *R* ^2^ for this function is shown. Representative images of transverse sections of a vascular bundle in the rachis or stipe of *P. pseudoaureum* (b) 5 cm from the apex, (c) 15 cm from the apex, and (d) 60 cm from the apex. Sections were stained with aqueous toluidine blue, and images were taken under visible light at ×10 magnification. The arrow in b shows the location of the xylem. Bars, 500 μm. Error bars show ±SE (*n* = 3).

No change in *Dh* was observed along the length of the measured terrestrial, prostrate rhizome of *P. pseudoaureum* (Fig. 4a). Mean rhizome *Dh* in *P. pseudoaureum* ranged from 83.07 μm at the rhizome apex to 82.28 μm 40 cm away from the apex (Table 1). *Dh* in the rhizome of the *P. pseudoaureum* was the largest of the species examined. By contrast, *Dh* declined from the base of the stipe towards the apex of the leaf in *P. pseudoaureum* (Fig. 5a). This decline in *Dh* across the stipe and into the midrib of the leaf lamina was pronounced, declining from 76.13 μm to 25.07 μm over the span of 55 cm across the frond (Fig. 5a). This tip‐to‐base widening could be explained by a significant two‐parameter power function (*R* ^2^ = 0.99, *P* < 0.001) with an exponent of 0.46 (Fig. 5a; Table 1).

### Water status

In *M. hirsuta* we observed that leaf water potential declined with proximity to the apex in rhizomes that grew out of standing water and into air (Table 2; Fig. 2b). Midday leaf water potential was −0.88 MPa at the apex, −0.53 MPa 20 cm from the apex and − 0.42 MPa 40 cm from the apex where the rhizome was in closest proximity to standing water (Table 2; Fig. 1b). Significant differences were also observed in leaf water potential across the vertical stems of *E. giganteum* (Fig. 3b) with a midday water potential of −1.79 MPa at the apex, but −0.51 MPa in side shoots 120 cm from the apex around the middle of the vertical stem (Table 2). We also observed a significant difference in stem water potential of −0.41 MPa at the apex and −0.12 MPa 120 cm from the apex in *E. giganteum* (Fig. 3a). No difference in water potential was observed in leaves taken from along the terrestrial, prostrate rhizome of *P. pseudoaureum* (Fig. 4b) that grew along the soil surface, with mean leaf water potential varying between −0.64 and −0.72 MPa (Table 2).

**Table 2:** Mean leaf water potential along the stem or rhizome.

Species	Distance from apex (cm)	Leaf water potential (MPa) ± SE
*Marsilea hirsuta*	1	−0.88 ± 0.04^a^
	20	−0.53 ± 0.01^b^
	40	−0.42 ± 0.03^c^
*Equisetum giganteum*	1	−1.79 ± 0.17^a^
	120	−0.51 ± 0.04^b^
*Phlebodium pseudoaureum*	1	−0.64 ± 0.1^a^
	20	−0.71 ± 0.03^a^
	40	−0.72 ± 0.01^a^

Different letters indicate significant differences in water potential across a rhizome or stem using a *post‐hoc* pairwise Tukey's test at *P* < 0.05.

## Discussion

Given the primary xylem in ferns, as well as the diversity of growth forms in this group of plants, from arborescent species to those with terrestrial or even aquatic rhizomes, ferns provide an excellent system in which to investigate whether tip‐to‐base conduit widening is adaptively relevant in land plants and to interrogate the mechanism driving this phenomenon. Our observations revealed that tip‐to‐base conduit widening in ferns manifests in rhizomes, stems, or leaves that require the maintenance of hydraulic efficiency along the full pathlength for water flow to the apex, and not in rhizomes or stems that, due to growth habit or environment, do not require long‐distance water transport. Our result suggests that the development of wider conduits away from the apex maintains efficient hydraulic supply in stems and leaves that require it (i.e. vertically growing stems), whereas when there is relaxed pressure on efficient hydraulic supply across the stem (i.e. in aquatic rhizomes or those that root at each node) then alternative scaling functions can manifest without lethal consequence. Interestingly, we found that the exponent of the power function of the relationship between *Dh* and distance from the apex was similar to a universal value reported widely across vascular plants in *M. hirsuta* or was higher in the stems of *E. giganteum* and the fronds of *P. pseudoaureum* (Table 1). It has been hypothesized that the exponent for this relationship will be higher in leaves so that hydraulic resistance is kept independent of leaf length to ensure that plasticity in leaf size does not influence whole plant hydraulic resistance (Lechthaler *et al*., 2020), which explains the considerably higher tip‐to‐base widening exponent in the fronds of *P. pseudoaureum* (Olson *et al*., 2021). Similarly, the stems of *E. giganteum* are also the primary photosynthetic organ bearing leaves that are appressed to the stem and numerous side shoots; the increase in size towards the base of the main stem may also serve to counter the increase in the total pathlength for water flow in this species, necessitating a higher exponent.

Most studies show that *Dh* increases away from the apex to the base of the stem (Zimmermann, 1983; Meinzer *et al*., 2001; Nijsse *et al*., 2001; Martínez‐Vilalta *et al*., 2002; McCulloh & Sperry, 2005; Koçillari *et al*., 2021), with many concluding that this is an adaptively relevant characteristic of all land plants (Petit, 2024); yet the mechanism driving this anatomical trait remains unknown. Our results suggest that water status might canalize the developmental potential of the xylem such that a universal scaling of conduit diameters towards the apex manifests. In *P. pseudoaureum* with a rhizome that roots at each node in direct contact with the soil and in the aquatic rhizomes of *M. hirsuta*, we found that there was no significant variation along the stem in *Dh*. In these species, leaf water potential remained high in the apex and did not vary across nodes (Table 2). By contrast, *Dh* declined towards the apex of both the vertical stems of *E. giganteum* (Fig. 3a–c), the non‐aquatic stems of *M. hirsuta* (Fig. 2a–c) and across the fronds of *P. pseudoaureum* (Fig. 5). Only in these tissues, in which hydraulic efficiency needed to be maintained, did we observe tip‐to‐base conduit widening (Table 2; Fig. 3a). Water potential gradients, as well as auxin levels, significantly affect the development of xylem in angiosperms (Aloni & Zimmermann, 1983; Brodribb & Hill, 2000; Johnson *et al*., 2018), from cell size to wall structure, presumably influencing xylem function and the hydraulic architecture of the individual. In terms of cell size, high turgor pressure appears to be key to driving maximum cell expansion in the meristem (Kazuyuki *et al*., 1997; Ali *et al*., 2022).

Our observation that the development of tip‐to‐base conduit widening occurs in aerial stems of *M. hirsuta* indicates that this developmental phenomenon has not been lost in aquatic species, but will only manifest under conditions that require the maintenance of hydraulic efficiency (Anfodillo & Olson, 2024). Whether these results are generalizable to all aquatic plants remains to be tested, but there are suggestions that an absence of tip‐to‐base conduit widening is common to the stems of numerous aquatic species (Olson pers. comm.). These results also highlight that there is considerable developmental potential in the xylem, but that a universal scaling of tip‐to‐base conduit widening occurs when a buffering of hydraulic resistance along the stem is required. Other studies have shown that there is considerable developmental potential in xylem anatomy regulated by hormones or genes (Aloni & Zimmermann, 1983; Johnson *et al*., 2018; Rodriguez‐Zaccaro *et al*., 2021), which might be the primary determinant of this anatomical change, although more work is required to determine the specific mechanism that drives tip‐to‐base conduit widening when organs require it.

The rhizomes of ferns growing on the surface of the soil and rooting at each node or in water, as is the case in *P. pseudoaureum* and the aquatic rhizomes of *M. hirsuta*, respectively, are able to maintain a constant water status across the rhizome irrespective of the number of nodes along the rhizome. Fern rhizomes that grow horizontally along the soil surface typically root at each node and distribute nutrients and water evenly across a wide area (Suissa *et al*., 2023). Lateral distribution of water rather than apical transport means that any tip‐to‐base widening would have no benefit for hydraulic efficiency in these species (Suissa *et al*., 2023). Suissa *et al*. (2023) have shown that the phytomers along the rhizomes of terrestrial ferns are hydraulically isolated, suggesting that long‐distance water transport along the rhizome is minimal and that the hydraulic rules governing tip‐to‐base conduit widening in arborescent stems might not apply to these plants. By contrast, if stems grow into drier environments or aboveground, tip‐to‐base conduit widening in these organs will mitigate severe declines in water potential towards the apex if widening did not occur.

Through the use of ferns, we show that tip‐to‐base conduit widening manifests in tissues and organs in which maximizing hydraulic efficiency is paramount; that is, in organs that receive water supply from a single, basal location. Our results suggest that in fern organs in which there is no apical water transport, there is no tip‐to‐base conduit widening. Our results suggest that under conditions in which selection does not favor tip‐to‐base conduit widening for hydraulic efficiency, then a relaxation of developmental potential occurs, whereby conduit diameters tend to approach a maximum rapidly. Our results indicate considerable developmental potential for xylem conduit diameter exists under conditions in which hydraulic efficiency is not required for survival, but that tip‐to‐base conduit widening consistently manifests in organs where there is a need to buffer the resistance of the conductive path as it becomes longer.

## Competing interests

None declared.

## Author contributions

YK collected and analyzed data and wrote the manuscript; SAMM devised the study and revised the manuscript.

## Disclaimer

The New Phytologist Foundation remains neutral with regard to jurisdictional claims in maps and in any institutional affiliations.

## Data Availability

All data are included in the manuscript.
